# Increased glutamate and glutamine levels and their relationship to astrocytes and dopaminergic transmissions in the brains of adults with autism

**DOI:** 10.1038/s41598-023-38306-3

**Published:** 2023-07-19

**Authors:** Masaki Oya, Kiwamu Matsuoka, Manabu Kubota, Junya Fujino, Shisei Tei, Keisuke Takahata, Kenji Tagai, Yasuharu Yamamoto, Hitoshi Shimada, Chie Seki, Takashi Itahashi, Yuta Y. Aoki, Haruhisa Ohta, Ryu-ichiro Hashimoto, Genichi Sugihara, Takayuki Obata, Ming-Rong Zhang, Tetsuya Suhara, Motoaki Nakamura, Nobumasa Kato, Yuhei Takado, Hidehiko Takahashi, Makoto Higuchi

**Affiliations:** 1 Department of Functional Brain Imaging, Institute for Quantum Medical Science, National Institutes for Quantum Science and Technology (QST), 4-9-1 Anagawa, Inage-ku, Chiba-shi, Chiba 263-8555 Japan; 2 Department of Psychiatry and Behavioral Sciences, Graduate School of Medical and Dental Sciences, Tokyo Medical and Dental University, Bunkyo-ku, Tokyo Japan; 3 Department of Psychiatry, Nara Medical University, Kashihara-shi, Nara Japan; 4 Department of Psychiatry, Graduate School of Medicine, Kyoto University, Kyoto-shi, Kyoto Japan; 5 Medical Institute of Developmental Disabilities Research, Showa University, Setagaya-ku, Tokyo Japan; 6 Institute of Applied Brain Sciences, Waseda University, Tokorozawa-shi, Saitama Japan; 7 School of Human and Social Sciences, Tokyo International University, Kawagoe-shi, Saitama Japan; 8 Department of Neuropsychiatry, Keio University School of Medicine, Shinjuku-ku, Tokyo Japan; 9 Center for Integrated Human Brain Science, Brain Research Institute, Niigata University, Niigata-shi, Niigata Japan; 10 Department of Psychiatry, School of Medicine, Showa University, Setagaya-ku, Tokyo Japan; 11 Department of Language Sciences, Graduate School of Humanities, Tokyo Metropolitan University, Hachioji-shi, Tokyo Japan; 12 Department of Molecular Imaging and Theranostics, Institute for Quantum Medical Science, National Institutes for Quantum Science and Technology, Chiba-shi, Chiba Japan; 13 Department of Advanced Nuclear Medicine Sciences, Institute for Quantum Medical Science, National Institutes for Quantum Science and Technology, Chiba-shi, Chiba Japan; 14 Kanagawa Psychiatric Center, Yokohama-shi, Kanagawa Japan; 15 Center for Brain Integration Research, Tokyo Medical and Dental University, Bunkyo-ku, Tokyo Japan

**Keywords:** Autism spectrum disorders, Positron-emission tomography, Magnetic resonance imaging

## Abstract

Increased excitatory neuronal tones have been implicated in autism, but its mechanism remains elusive. The amplified glutamate signals may arise from enhanced glutamatergic circuits, which can be affected by astrocyte activation and suppressive signaling of dopamine neurotransmission. We tested this hypothesis using magnetic resonance spectroscopy and positron emission tomography scan with ^11^C-SCH23390 for dopamine D1 receptors in the anterior cingulate cortex (ACC). We enrolled 18 male adults with high-functioning autism and 20 typically developed (TD) male subjects. The autism group showed elevated glutamate, glutamine, and myo-inositol (mI) levels compared with the TD group (*p* = 0.045, *p* = 0.044, *p* = 0.030, respectively) and a positive correlation between glutamine and mI levels in the ACC (r = 0.54, *p* = 0.020). In autism and TD groups, ACC D1 receptor radioligand binding was negatively correlated with ACC glutamine levels (r =  − 0.55, *p* = 0.022; r =  − 0.58, *p* = 0.008, respectively). The enhanced glutamate-glutamine metabolism might be due to astroglial activation and the consequent reinforcement of glutamine synthesis in autistic brains. Glutamine synthesis could underly the physiological inhibitory control of dopaminergic D1 receptor signals. Our findings suggest a high neuron excitation-inhibition ratio with astrocytic activation in the etiology of autism.

## Introduction

Autism is a developmental condition characterized by deficits in social communication and interaction, restricted interest, and repetitive behaviors. Autism has been associated with a high rate of suicide^1^ and impaired quality of life^2^. A survey in 2018 revealed that the autism spectrum disorder prevalence was 2.3% higher than previously reported^3^. Despite these critical issues, drug development to treat autism remains challenging^4^; understanding the pathophysiology of autism at the cellular and neurochemical levels is needed for finding novel targets to develop therapeutic treatments.

In the brain, assemblies of excitatory and inhibitory neurons are involved in learning and memory and the balance between their activities is critically significant for their network development^5^. One of the plausible theories for the pathophysiology of autism is the excitation-inhibition (E/I) imbalance theory^6^. Accordingly, increased E/I ratio has been implicated in autism^7,8^. At a non-clinical level, a mouse model of autism was reported to exhibit an elevated excitatory synaptic input in comparison to the inhibitory synaptic inputs^9,10^. In addition, optogenetic enhancement of the E/I ratio in the mouse neocortex provoked deficits in social behaviors relevant to autism^11^. In humans, disruption of inhibitory circuits putatively raised the E/I ratio as shown in electroencephalography^12^.

Magnetic resonance spectroscopy (MRS) allows to investigate the balance between the excitatory and inhibitory neuronal activities in the brains of living subjects, as this technology allows measuring excitatory glutamate (Glu), its relative metabolite glutamine (Gln), and the inhibitory gamma-aminobutyric acid (GABA) concentrations^13–15^. Only three studies which attempted to assay Glu and Gln levels separately by the conventional point resolved spectroscopy (PRESS) protocol in subjects with autism^16–18^. Among them, the two studies showed alterations in Glu^18^ and Gln^16^ levels in the anterior cingulate cortex (ACC). In contrast to conventional MRS protocols^16–18^, advanced methods offer more precise and sensitive detection of brain metabolites^19^, but they have so far not been applied to autism. Recently, we have demonstrated that a short-echo time (TE) spin-echo full-intensity acquisition localized single voxel spectroscopy (SPECIAL) sequence could robustly assess Gln levels in the brain, which correlated with plasma levels^20^. Accordingly, this approach might facilitate investigating the existence of an altered E/I ratio in the ACC of subjects with autism.

Although the mechanism underlying the augmented excitatory neuronal tone in autism is yet to be clarified, several lines of evidence support that astrocytes are involved in the modulation of the neuronal excitability in central nervous system diseases^21,22^, as astrocytes are involved in the regulation of Glu, Gln, and GABA levels in the brain^23^. For instance, Glu is converted to Gln by glutamine synthetase (GS), an enzyme found in astrocytes in the mammalian brain^24^. In fact, some studies have highlighted the contribution of astrocytes to the pathophysiology of autism^25^. Alternatively, human studies reported changes in the expression of astrocytic markers in postmortem brains as exemplified by increased glial fibrillary acidic protein (GFAP) and connexin 43 and decreased aquaporin 4 levels^26^. As such, elevated GFAP levels were detected in plasma samples derived from autistic patients^27^. Moreover, astrocytic modification of excitatory neurotransmitters is mechanistically linked to the dopaminergic system. Mice lacking dopamine (DA) D1 receptors (Rs) displayed increased Gln levels in the brain, an effect ameliorated by L-DOPA administration^28^. Moreover, autistic behaviors are hypothesized to arise from dopaminergic dysfunction^29^. Our recent study using positron emission tomography (PET) revealed the association between DA D1R radioligand binding in several brain regions with characteristic symptoms of adults with autism^30^. Therefore, DA signals mediated by D1Rs might be involved in aberrant Glu-Gln cycles and the consequent dysregulation of the neuronal E/I balance in autism. Despite these indications, no studies using in vivo imaging of human brain tissues have examined astrocyte activation, changes in the E/I balance in autism and its relationship with the dopaminergic nervous system.

Herein, we aimed at examining the hypothesis stating that the excitatory glutamatergic neurotransmissions were enhanced in the autistic brains in association with the astrocytic activation which is related to Gln synthesis and disrupted dopaminergic signals through D1Rs. Toward this end, we quantified Glu, Gln, and GABA levels by MRS to evaluate the excitatory versus the inhibitory neuronal tones, along with MRS assays of myo-inositol (mI), a marker for astrocytic activation. We also investigated the inter-metabolic correlations between mI and Gln levels. Therefore, we measured the metabolite levels and the regional DA D1R bindings with a specific PET radioligand of ^11^C-SCH23390. We focused on the ACC for its disrupted structural^31,32^ and functional^33,34^ properties as evidenced in the autistic brain. Participants were limited to adults with high-functioning autism, and males considering a potential gender difference in the E/I ratio^35^.

## Results

### Participant characteristics

As previously mentioned, participants’ demographic and clinical characteristics are summarized in Table 1.

**Table 1:** Summary of demographic and clinical characteristics of individuals with autism and TD.

	Autism (N = 18)		TD (N = 20)		Statistics	
	Mean	SD	Mean	SD	t/χ^2^	*p*
Age (years)	33.1	7.7	30.1	5.9	t = 1.34	0.19
Education (years)	15.3	1.8	15.1	1.5	t = 0.43	0.67
IQ	103.7	16.4	105.8	8.1	t = − 0.48	0.64
Handedness (right/left)	16/2		18/2		χ^2^ = 0.01	1.00
AQ	32.0	5.9	N/A		N/A	
Social skill	7.1	2.2				
Attention switching	6.6	2.1				
Attention to detail	4.7	2.1				
Communication	6.9	2.1				
Imagination	6.7	2.2				
Injected radioactivity for ^11^C-SCH23390 (MBq)	225.7	6.7	224.7	7.1	t = 0.44	0.67
Molar activity for ^11^C-SCH23390 (GBq/μmol)	63.6	17.2	69.4	16.8	t = − 1.06	0.30

*AQ* Autism spectrum quotient; *SD* Standard deviation; *IQ* Intelligence quotient; *TD* Typically developed.

*p < 0.05.

### Comparisons of metabolite levels between individuals with autism and typically developed (TD)

**Figure 1:**
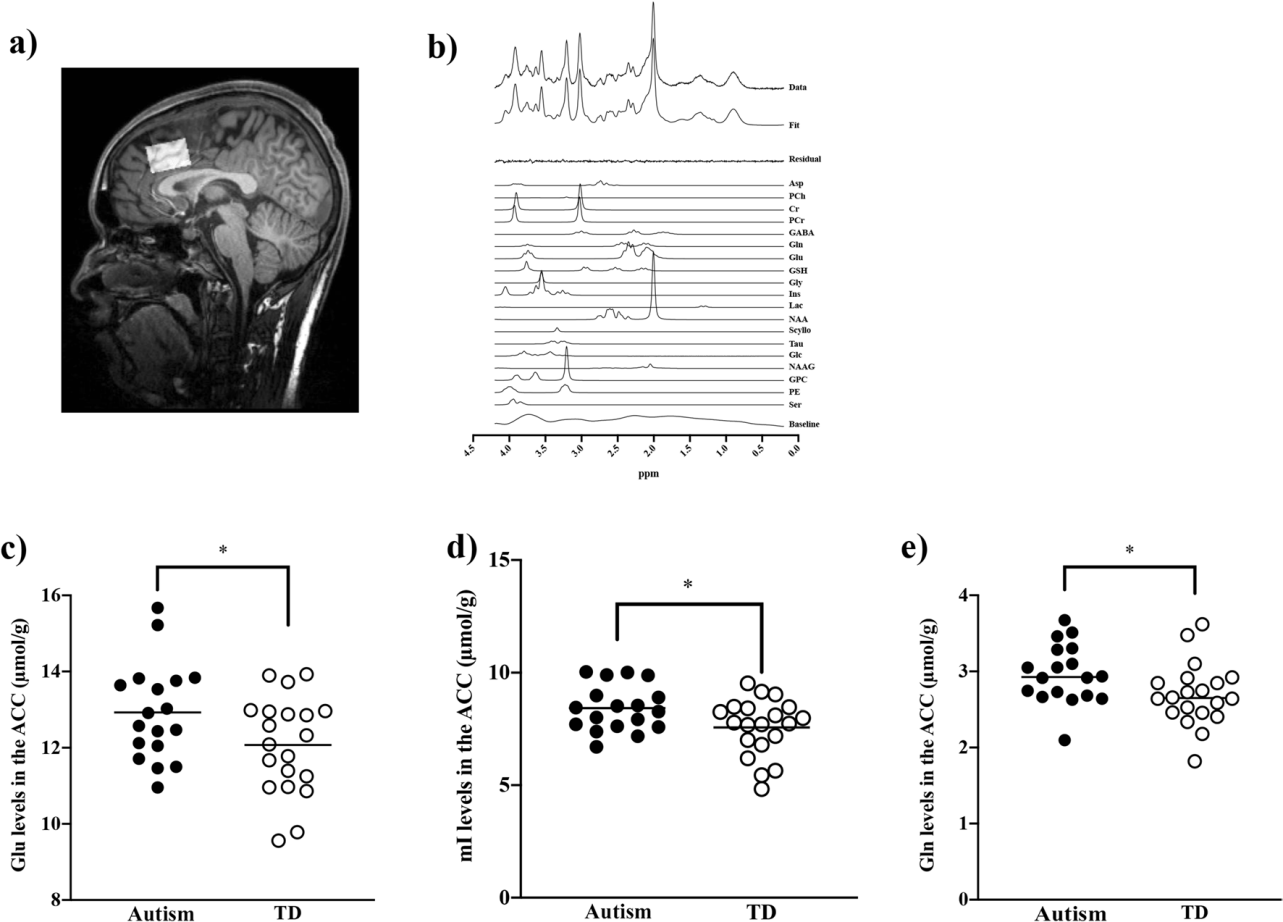
Representative MRS spectrum and VOIs and scatterplots of metabolite levels in the ACC of subjects with autism and TD. (**a**) ACC VOI (30 × 20 × 20 mm^3^) of a TD individual. (**b**) Example of a MRS spectrum. (**c**) Glu, (**d**) Gln, and (**e**) mI levels in the ACC were significantly increased in autism compared with TD (Glu, *p* = 0.045; Gln, *p* = 0.044; mI, *p* = 0.030). ACC, anterior cingulate cortex; GABA, γ-aminobutyric acid; Gln, glutamine; Glu, glutamate; mI, myo-inositol; TD, typically developed; VOI, volume of interest. *p < 0.05.

Glu levels were significantly increased in the ACC in adults with autism compared to TD subjects (12.93 [1.27] for the autism group, 12.07 [1.26] for the TD group, t = 2.08, *p* = 0.045), Gln (2.97 [0.39] for the autism group, 2.70 [0.41] for the TD group, t = 2.09, *p* = 0.044), and mI (8.42 [1.03] for the autism group, 7.57 [1.27] for the TD group; t = 2.26, *p* = 0.030) (Fig. 1). Alternatively, we found no significant differences in ACC GABA levels (2.38 [0.31] for the autism group, 2.29 [0.40] for the TD group; t = 0.79, *p* = 0.44).

### Correlations of Gln levels with mI levels and clinical measures

**Figure 2:**
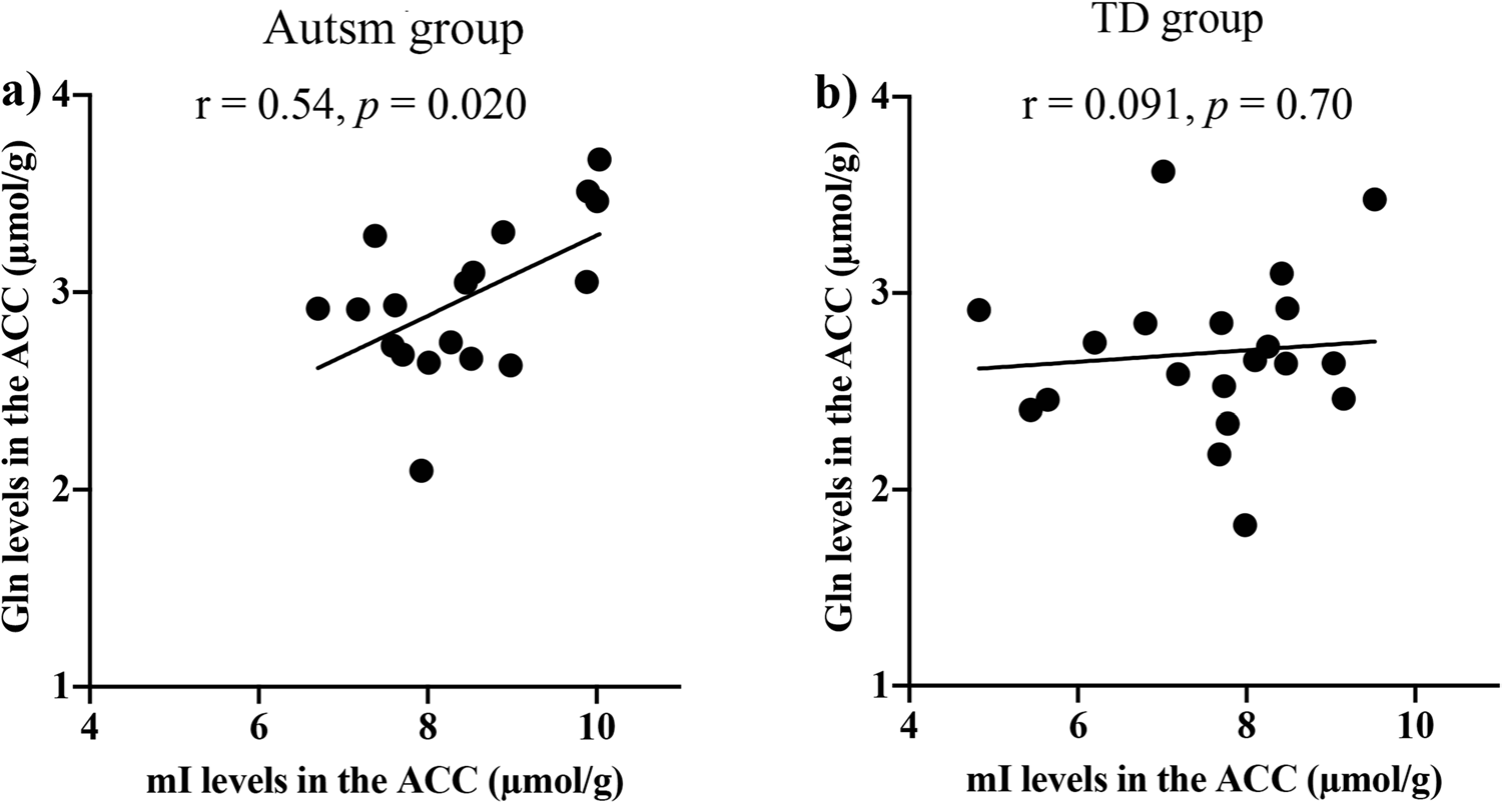
mI and Gln levels in the ACC of subjects with autism and TD. (**a**) mI and Gln levels in the ACC were significantly correlated (r = 0.54, *p* = 0.020) in individuals with autism. (**b**) Conversely, no correlation was found in individuals with TD (r = 0.091, *p* = 0.70). ACC, anterior cingulate cortex; Gln, glutamine; mI, myo-inositol; TD, typical developed. *p < 0.05.

**Figure 3:**
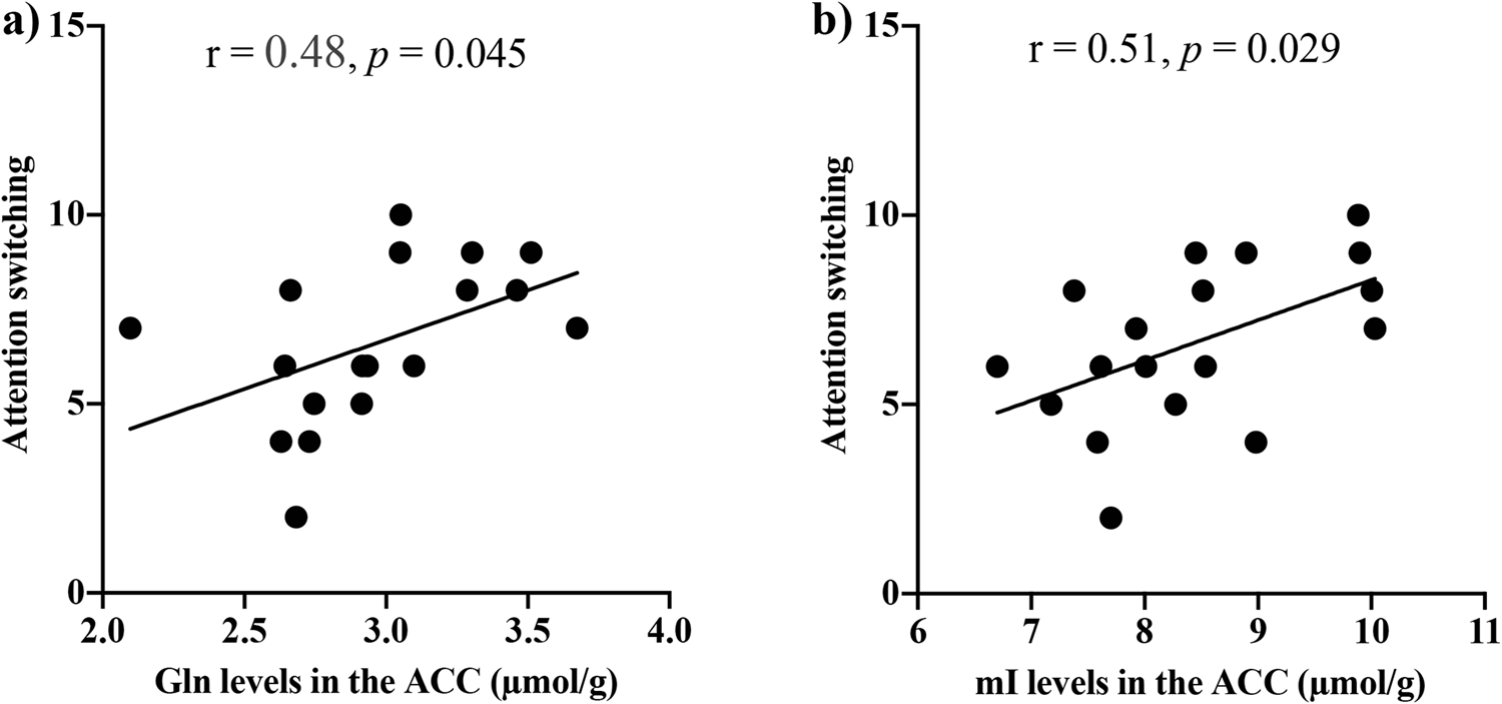
AQ Attention switching subscale score with Gln and mI levels in the ACC of individuals with autism. The AQ Attention switching subscale score was significantly positive correlated with (**a**) Gln levels (r = 0.48, *p* = 0.045) and with (**b**) mI levels (r = 0.51, *p* = 0.029) in the ACC in individuals with autism. ACC, anterior cingulate cortex; AQ, autism spectrum quotient; Gln, glutamine; mI, myo-inositol. *p < 0.05.

We found significantly positive correlations between mI and Gln levels in the ACC of individuals with autism (r = 0.54, *p* = 0.020) but not in TD subjects (r = 0.091, *p* = 0.70) (Fig. 2). In addition, we found significantly positive correlations between Autism Spectrum Quotient (AQ)^36^, Attention switching subscale score, and Gln (r = 0.48, *p* = 0.045) and mI levels (r = 0.51, *p* = 0.029) in the ACC in individuals with autism (Fig. 3, Table S1).

### ***Correlations between DA D***_***1***_***R binding and metabolite levels***

**Figure 4:**
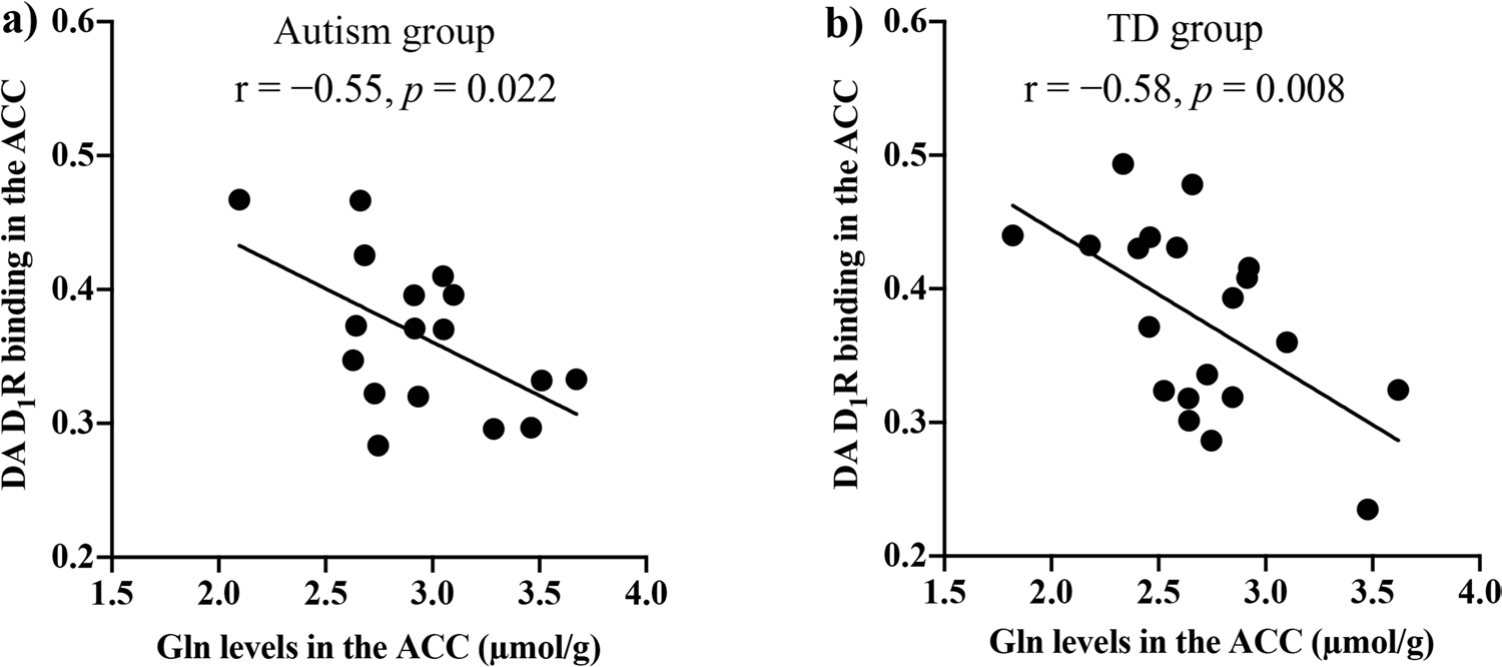
Correlations of DA D_1_R binding with Gln levels in the ACC in individuals with autism and TD. Significant negative correlations between DA D_1_R binding and Gln levels in the ACC of individuals with (**a**) autism (r =  − 0.55, *p* = 0.022) and (**b**) TD (r =  − 0.58, *p* = 0.008). ACC, anterior cingulate cortex; DA D_1_R, dopamine D_1_ receptor; Gln, glutamine; TD, typical developed. *p < 0.05.

As reported previously^30^, there were no significant differences in radioligand binding to DA D_1_Rs between groups (0.36 [0.06] for the autism group, 0.38 [0.07] for the TD group). On the other hand, there was a negative correlation between DA D_1_R binding and Gln levels in the ACC (r =  − 0.55, *p* = 0.022) of subjects with autism (Fig. 4). We also found negative correlations of Gln levels with DA D_1_R binding in the ACC (r =  − 0.58, *p* = 0.008) of TD subjects (Fig. 4). In contrast, there were no significant correlations of DA D_1_R binding in any of these regions with mI and Glu levels in the ACC of any participants (*p* > 0.05) (Table S2).

## Discussion

In this study, we found elevated Glu, Gln, and mI levels in the ACC of adults with autism compared with TD subjects. Subsequently, we found a positive correlation between Gln and mI levels in the ACC of subjects with autism but no correlation in TD subjects. ^11^C-SCH23390 PET also indicated that DA D1R binding was negatively correlated with Gln levels in the ACC in both adults with autism and TD subjects, but not with mI levels. With respect to clinical symptoms, subjects with autism showed a correlation between AQ Attention switching subscale scores and Gln and mI levels in the ACC.

The present study revealed that adults with autism exhibit increased excitatory Glu and Gln levels in the ACC. Our findings are supported by the evidence of neural hyperexcitability in autism^7,8^. While numerous MRS studies have attempted to clarify the E/I balance in autism^15^, only three separately evaluated Glu and Gln levels^16–18^. One reported increased Gln levels in the ACC^16^ while the others showed decreased Glu levels in the ACC^18^ and the striatum^17^. However, the SPECIAL sequence allowed us to evaluate Glu, Gln, and GABA levels separately and concurrently^37^. This advanced MRS protocol provided evidence of elevated levels of both excitatory neurotransmitters Glu and Gln in the ACC. As another feature of our study, the limited samples of male individuals with autism might contribute to revealing increased excitatory neurotransmitters, given the sex difference in the excitatory-inhibitory ratio in individuals with autism^35^. The relationship between autism and E/I imbalance has also received attention from a mechanistic viewpoint in genetic disorders. In fact, Fragile X syndrome, which is caused by changes in a single gene and is associated with autistic manifestations^38,39^, has shown aberrant E/I ratios according to several lines of reports (reviewed in^40^) in agreement with the present findings.

Consistent with previous studies^41,42^, adults with autism showed an increase in levels of mI^43^. Based on accumulated evidence from animal^44,45^ and human studies^26,27^ concerning the role of astrocytes in autism, altered astroglial functions have been hypothesized in order to contribute to the pathogenesis of autism^25^. Under metabolic homeostasis, astrocytes play a pivotal role in Gln synthesis via an astrocyte-specific enzyme, the GS^24^. Correspondently, the increased mI levels correlated with Gln levels in the ACC of individuals with autism. This might reflect that reactive astrocytes might produce higher Gln amounts in the brain of adults with autism; however, MRS-measured Gln levels may not solely reflect Gln in astrocytes. The disrupted homeostasis of the Glu-Gln cycle caused by astrocyte-specific Glu transporter (GLT1) deficiency was reported to induce pathological repetitive behaviors; this autistic-like behavior was alleviated by NMDA receptor inhibitors^46^. In addition, a human study reported increased GS plasma levels in individuals with autism^47^. These studies suggest that the disrupted homeostasis of the Glu-Gln cycle induced by reactive astrocytes might be associated with the pathology of autism. In the present study, we also observed a correlation between AQ Attention switching subscale scores and Gln and mI levels in the ACC, where neural activity is associated with attentional control^48^. Collectively, reactive astrocytes might be associated with increased excitatory balance, which may have potential applications for the development of drug treatments for autism^49^.

**Figure 5:**
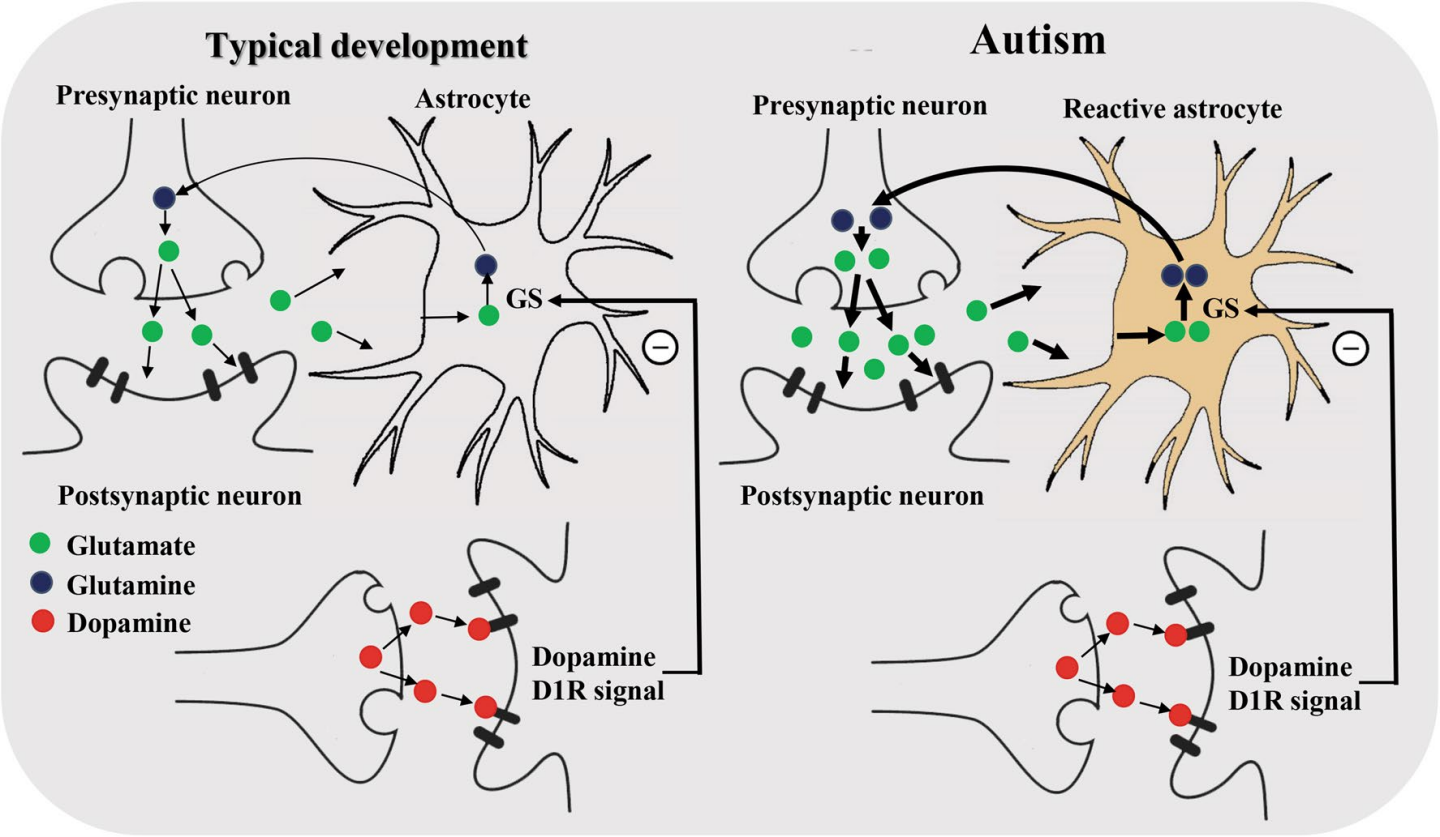
Summary of the enhanced Glu-Gln metabolism in autism. In physiological conditions, astrocytes absorb the Glu released from presynaptic neurons, where it is synthesized to Gln by GS. Subsequently, the synthesized Gln is transferred to presynaptic neurons and converted to Glu. In this Glu-Gln cycle, we speculate that DA D_1_R signals control Gln synthesis from Glu. Moreover, the Glu-Gln metabolism might be enhanced due to astrocyte activation in the autistic brain. DA D_1_R, dopamine D_1_ receptor; Gln, glutamine; Glu, glutamate; GS, glutamine synthetase.

On the other hand, we found that both adults with autism and TD subjects exhibited a negative correlation of DA D1R bindings with Gln levels in the ACC. Our findings indicated that DA D1R activation was involved in Gln synthesis (Fig. 5). Some animal studies suggested a regulatory role of dopaminergic neurons in Gln synthesis^28,50,51^. Rodrigues et al.^28^ reported that blocking DA D1R or DA depletion in mice augmented Gln levels. Another study documented that DA denervation by MPTP induced elevated Gln levels accompanied by increased GS activity^50^. Moreover, DA D1R agonists can inhibit the ionotropic glutamate receptor, α-amino-3-hydroxy-5-methyl-4-isoxazolepropionic^51^. We speculated the existence of physiological mechanisms of inhibition of Gln synthesis related to dopaminergic D1R signaling in the ACC of adults with autism and TD subjects. In the autistic brain, reactive astrocytes might intensify Gln synthesis independently of the inhibitory role of dopaminergic neurons.

This study has some limitations. First, it focused on the ACC but did not investigate other brain regions, based on the assumption of its involvement in the pathology of autism. Further research is needed to comprehensively understand the relationship between autism and all brain regions. Second, some data could not withstand corrections for multiple comparisons because diverse metabolites and neurotransmission components were analyzed in comparative and correlational fashions. Therefore, our findings should be interpreted with caution regarding this point. Finally, we did not evaluate differences in adaptive functioning among the autistic disorder, Asperger syndrome, and TD groups. Such investigations would be performed in an additional study with an expanded sample size, given that individuals with Asperger syndrome exhibit impaired social adaptive skills.

In conclusion, the present study indicated an excessive E/I balance accompanied by increased Gln levels, associated with reactive astrocytes in the ACC of individuals with autism. Under the physiological inhibitory control of Gln synthesis by dopaminergic signaling, the enhanced Glu-Gln metabolism induced by reactive astrocytes might be one pathological factor responsible for the E/I imbalance found in autism. Our findings lend further evidence to the role of astroglial activity in Glu-Gln metabolism, its relationship with attention switching, and the inhibitory role of dopamine receptors in Gln synthesis.

## Materials and methods

### Participants

The participants’ demographic profiles are summarized in Table 1^30^. Briefly, we evaluated 18 adults with autism and 20 TD individuals with matching age, intelligence quotient (IQ), and handedness. All subjects underwent MRS and PET scans. All participants were adult males, nonsmokers, and not exposed to monoamine-acting drugs. The TD group ranged in age from 20 to 42 years, while the autism group ranged in age from 23 to 46 years. The participants were volunteers diagnosed with autism at the outpatient department of Showa University Karasuyama Hospital. Both participants and caregivers, having relevant information about participants’ early childhood, were interviewed for ~ 3 h by > 3 experienced psychiatrists and one clinical psychologist using the Diagnostic and Statistical Manual of Mental Disorders, fourth edition text revision (DSM-IV-TR) based on the following criteria: patient’s developmental history, illness presence, life history, and family history. The final diagnosis of autistic or Asperger syndrome was made based on the consensus among the psychiatrists and the clinical psychologist. The autism group consisted of 16 adults with autistic disorder and two with Asperger syndrome. No individual was diagnosed with a pervasive developmental disorder not otherwise specified (PDD-NOS) in the present study. According to the Structured Clinical Interview for DSM-IV Axis I Disorders (SCID), none of the participants with autism met the diagnostic criteria for substance use disorder, bipolar disorder, or schizophrenia.

TD subjects were recruited from the general population via advertisements and word-of-mouth. According to the SCID, none of these individuals met the diagnostic criteria for any psychiatric disorders and had no family history of serious medical or surgical illness, neurodevelopmental diseases, substance abuse, or neurodevelopmental diseases.

This study was approved by the Radiation Drug Safety Committee and the Institutional Review Board of the former National Institutes for former Quantum and Radiological Science and Technology, Japan, and by the institutional review board of Showa University Karasuyama Hospital, Japan. This study was conducted in accordance with the World Medical Association Code of Ethics. Written informed consent was obtained from all participants after providing them with a complete description of the study.

### Clinical assessments

Prior to the bioanalytical study, the IQ levels of adults with autism were evaluated by the Wechsler Adult Intelligence Scale-Third Edition (WAIS-III). Since IQ scores were > 75 points in all adults with autism, we considered they had high-functioning autism. There was no difference in IQ between the autistic disorder and Asperger syndrome groups (mean [SD], 102.8 [17.1] in the autistic disorder group versus 111.0 [7.1] in the Asperger syndrome group; t =  − 0.65, *p* = 0.52) (Fig. S1). Meanwhile, the IQ levels of individuals with TD were assessed with the Japanese version of the National Adult Reading Test (JART), which is reported to predict the full IQ score scale^52^. We assessed the autistic traits of adults with autism using AQ^36^.

### Magnetic resonance imaging (MRI) and MRS data acquisition

We performed all MRI and MRS tests using a 3 T scanner (Siemens MAGNETOM Verio, Erlangen, Germany) with a 32-channel receiving head coil. The 3D volumetric acquisition of a T1-weighted gradient-echo sequence produced a gapless series of thin sagittal sections (repetition time (TR) = 2,300 ms; TE = 1.95 ms; inversion time (TI) = 900 ms; field of view (FOV) = 250 mm; flip angle = 9°; acquisition matrix = 256 × 256; axial slices thickness = 1 mm). To determine the voxels of interest (VOIs) localized to the ACC, we used anatomical images (Fig. 1).

As previously described, we applied MRS on the ACC using a special sequence^37^ with the following parameters^20^: TE = 8.5 ms, TR = 3000 ms, average number = 128, and VOI dimensions = 30 × 20 × 20 mm^3^. After running a 3D shim (Syngo MR version for B17, Siemens, Erlangen, Germany), we performed a manual shim so that the line width of the water spectrum in magnitude mode was < 20 Hz. Prior to the SPECIAL localization sequence, we applied an outer volume and water suppression with variable-pulse power and optimized relaxation delays^53^. We computed the tissue composition within the VOI based on 3D T1-weighted image segmentation using Gannet3.0^54^. We calculated the water concentration in the white matter (WM), gray matter (GM), and cerebrospinal fluid (CSF) for the spectral analysis using a linear combination model (LCModel)^55^ based on the volume fractions of these segments. The concentrations were 35,880 mM, 43,300 mM, and 55,556 mM, respectively. We obtained the signal-to-noise ratio (SNR) by dividing the peak height of N-acetyl aspartic acid (NAA) at 2.01 ppm by the standard deviation (SD) of noise. For all spectra, we performed the LCModel quantification in a spectral window of 0.2–4.2 ppm. Macromolecules (MM) were fitted using LCModel’s default parameterized MM resonances while using the default LCModel baseline parameters.

### MRS data analysis

We applied a weighted combination of receiver channels, followed by motion corruption average removal, spectrum registration for frequency and phase drift correction, and pre-subtraction sub-spectral alignment in MATLAB 2019a (The Mathworks, Natick, MA, USA) using the FID-A toolkit before signal averaging and data analysis^56^. To analyze MRS data, we used the LCModel with a basis set of simulated spectra comprising the following 20 metabolites: Aspartate (Asp), phosphocholine (PCh), creatine (Cr), phosphocreatine (PCr), GABA, Gln, Glu, glutathione (GSH), glycine (Gly), mI, lactate (Lac), NAA, scyllo-inositol (Scyllo), taurine (Tau), glucose (Glc), N-acetylaspartylglutamate (NAAG), glycerophosphocholine (GPC), phosphorylethanolamine (PE), Serine (Ser), and MM (Fig. 1). Among these metabolites, we focused on GABA, Gln, and Glu to evaluate the E/I ratio and on mI as an astrocyte marker^43,57^. The spectral SNR and the linewidth (LCModel output) in the ACC for the subjects with autism and TD were 76.6 ± 9.1 and 0.028 ± 0.004 ppm, respectively. We corrected the metabolite levels by segmenting GM, WM, and CSF in the VOI^58^.

### PET procedures

We used the same procedures as in our previous study regarding PET data^30^. Each participant underwent a PET scan with ^11^C-SCH23390^59^ to visualize DA D1Rs. All PET scans were carried out with a Biograph mCT flow system (Siemens Healthcare, Erlangen Germany) which provides 109 transaxial sections with an axial FOV of 21.8 cm. The intrinsic in-plane and axial spatial resolutions yielded by this scanner are 5.9 and 5.5 mm full-width at half-maximum. Prior to the emission data, we conducted a computed tomography (CT) scan for attenuation correction. To minimize participants’ head movement during PET measurements, we used a head fixation device. However, one person with autism was excluded from PET analysis due to significant intra-frame motion. In addition, due to fatigue complaints, the ^11^C-SCH23390 PET scan was terminated at 52 min after radiotracer injection in one subject with TD.

We acquired a list-mode data with a PET-CT system for 60 min immediately after intravenous rapid bolus injection of ^11^C-SCH23390. Then, we sorted and rebinned the data with 38-frame signatures increasing from 20 s to 4 min (20 s × 12, 1 min × 16, and 4 min × 10). We corrected the sinograms for attenuation using the CT images, using the delayed coincidence counting method for random samples and the single-scatter simulation method for scatter samples. Subsequently, we reconstructed the modified sinograms using a filtered back-projection algorithm with a Hanning filter (4.0 mm full-width at half-maximum).

### PET and MRI data processing

We performed co-registration of motion-corrected PET images with the corresponding individual’s T1-weighted MRI using PMOD® software ver. 3.8 (PMOD Technologies Ltd, Zurich, Switzerland). We used FreeSurfer software (version 6.0.0; http://surfer.nmr.harvard.edu) for surface-based cortical reconstruction and volumetric subcortical segmentation for each T1-weighted image. We defined regions of interest (ROIs) using brain atlases^60–62^. ^11^C-SCH23390 binding to DA D1Rs was quantified as nondisplaced tissue (*BP*_ND_). Next, we calculated the *BP*_ND_ for the target ROI in the ACC using a three-parameter simplified reference tissue model^63^ with the cerebellar cortex excluding the vermis as a reference tissue. ROIs were found to be similar to our previous study^30^.

### Statistical analysis

The results are indicated as mean and SD (mean [SD]). We applied independent sample t-tests and χ^2^-tests for the statistical examination for all participants regarding differences in demographics, voxel-wise MRS data, AQ scores, and regional PET radioligand binding. To test correlations between MRS data, AQ subscale scores, and PET radioligand binding, we performed Pearson correlation and Spearman's partial rank-order correlation analyses. The latter was applied for AQ communication and imagination subscale scores, since they are not normally distributed. Statistical analyses were conducted with IBM SPSS Statistics for Windows, version 25 (IBM Corp., Armonk, N.Y., USA). The statistical significance threshold was set at *p* < 0.05 (two-tailed) for group comparisons and correlation analyses.

## Supplementary Information

Supplementary Information.

## Data Availability

Data can be obtained upon reasonable request. Anonymized raw data supporting the findings of the present study may be shared upon request with the corresponding author.
