# Supplementary material for: Increased glutamate and glutamine levels and their relationship to astrocytes and dopaminergic transmissions in the brains of adults with autism
Source: Sci Rep. 2023 Jul 19;13:11655. doi: 10.1038/s41598-023-38306-3 (PMC10356952; doi:10.1038/s41598-023-38306-3)
Supplement: Supplementary file 1 — Supplementary Information. [file 41598_2023_38306_MOESM1_ESM.pdf]

Supplementary Tables

Table S1. Correlations of metabolite levels in the ACC with AQ scores in individuals with autism

	AQ									
	Social skill		Attention switching		Attention to detail		Communication		Imagination	
	r	*p*	r	*p*	r	*p*	r	*p*	r	*p*
GABA	0.21	0.40	0.35	0.15	−0.15	0.57	0.30	0.24	−0.32	0.19
Gln	−0.16	0.54	0.48	0.045*	0.22	0.38	0.39	0.11	0.14	0.59
Glu	−0.15	0.56	0.42	0.081	−0.027	0.92	0.36	0.14	0.18	0.47
mI	0.006	0.98	0.51	0.029*	0.062	0.81	0.17	0.51	0.073	0.77

AQ, autism spectrum quotient; GABA, γ-aminobutyric acid; Gln, glutamine; Glu, glutamate; mI, myo-inositol. * $p < 0.05$.

Table S2. Correlations of metabolite concentrations with DA D1R binding in the ACC of individuals with autism and TD

	DA D1R binding	
	r	*p*
**Autism**		
GABA	0.077	0.77
Gln	−0.55	0.022*
Glu	−0.15	0.56
mI	−0.20	0.45
**TD**		
GABA	−0.43	0.056
Gln	−0.58	0.008 *
Glu	−0.32	0.17
mI	−0.15	0.52

ACC, anterior cingulate cortex; DA D1R, dopamine D1 receptor; GABA, γ-aminobutyric acid; Gln, glutamine; Glu, glutamate; mI, myo-inositol; TD, typical developed. * $p < 0.05$.

Supplementary Figure

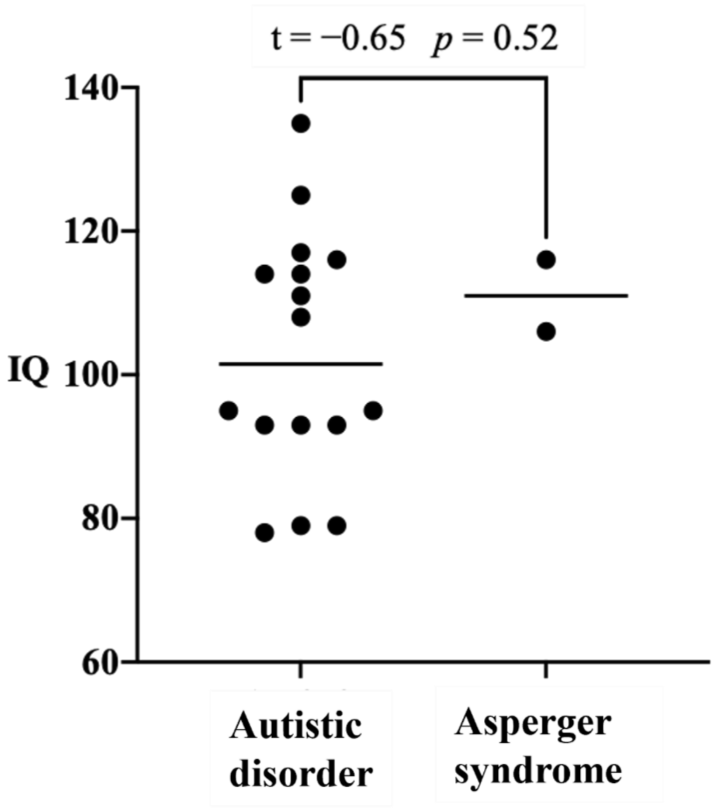

Figure S1. Scatterplot of IQ for subjects with autistic disorder and Asperger syndrome

All study participants had IQs above 75. In the present study, 16 adults with autistic disorder and two adults with Asperger syndrome were enrolled. There was no significance in IQ between the two groups (mean [SD], 102.8 [17.1] in the autistic disorder group versus 111.0 [7.1] in the Asperger syndrome group; t = −0.65, p = 0.52).
